# Uterine Leiomyosarcoma in a Young Woman: A Report of a Rare Case

**DOI:** 10.7759/cureus.63116

**Published:** 2024-06-25

**Authors:** Sakshi Heda, Preeti Gattani, Ayush Heda, Rasika D Zade, Rajesh G Gattani

**Affiliations:** 1 Obstetrics and Gynaecology, All India Institute of Medical Sciences, Rishikesh, Rishikesh, IND; 2 Obstetrics and Gynaecology, Datta Meghe Medical College, Datta Meghe Institute of Higher Education and Research (Deemed to be University), Nagpur, IND; 3 General Surgery, Jawaharlal Nehru Medical College, Datta Meghe Institute of Higher Education and Research (Deemed to be University), Wardha, IND

**Keywords:** leiomyoma, myomectomy, nulliparous, infertility, uterine sarcomas, : leiomyosarcoma

## Abstract

Uterine leiomyosarcoma (LMS) is an uncommon disease that arises from the smooth muscles present in the uterus. It usually occurs in post-menopausal women. Due to its aggressive nature, it has a very poor prognosis. We present a case of uterine LMS, which presented at a young age of 35 years for infertility, which is rare at this age. She had a fundal fibroid for which myomectomy was done. On histopathology, she was diagnosed with LMS. It is very difficult and nearly impossible to diagnose LMS preoperatively by available imaging modalities. There is an urgent need for a reliable preoperative risk scoring system that can help in diagnosing malignancy so that a correct surgical pathway and treatment can be offered to patients. A total abdominal hysterectomy (TAH) with bilateral salpingo-oophorectomy (BSO) was done and was advised adjuvant chemotherapy. The patient remained disease-free and was put on chemotherapy.

## Introduction

Uterine sarcomas account for 4 to 9% of all invasive uterine malignancies. Their yearly incidence rate is less than two per one million females. The average age at diagnosis for leiomyosarcoma (LMS) is between 48 and 54 years. Uterine sarcomas have generally an aggressive clinical behavior, with a great tendency of local recurrence and distant spread [1]. Uterine LMS is a rare uterine malignancy with an annual incidence of 0.64 per 100,000 women [1]. It accounts for 1% of all uterine malignancies and approximately 30% of all uterine sarcomas [2]. It has a predilection for perimenopausal women with a median age of 50 years [3]. It is an extremely aggressive malignancy associated with a poor overall prognosis [4]. Presenting symptoms are vague and nonspecific. Computed tomography (CT), magnetic resonance imaging (MRI), and ultrasound cannot reliably and accurately distinguish between benign leiomyoma and LMS. Therefore, LMS is often diagnosed following surgical excision of a presumed benign uterine neoplasm. The risk that a woman of reproductive age will be diagnosed with uterine myoma once in her lifetime is between 20 and 40% [5]. There is a strong need for a reliable pre-operative risk score for LMS, in order to justify diagnostic means beyond clinical routine and to choose the correct surgical pathway [6].

Tumor grade and stage, according to the Federation of Gynaecology and Obstetrics, are the most important determinants for the prognosis and survival of such patients [1,7,8]. Other prognostic factors are the age at which diagnosis is made, the size of the tumor, mitotic rate, lympho-vascular invasion, menopausal status, and parity [9]. We present an unusual case of a nulliparous woman with uterine LMS, who presented with infertility at the young age of 35 years.

## Case presentation

A nulliparous woman of 35 years reported to the Outpatient Department as a case of 15 years of primary infertility and a lump in the abdomen. The patient was asymptomatic two years back when she noticed a mass in her lower abdomen. She did not have any menstrual complaints like menorrhagia, pain in the abdomen, or weight loss. Her menses were regular with average flow. The patient was thin-built, and her vitals were normal. Per-abdominal examination showed the presence of a lump around 28 weeks in size. The mass was smooth, firm, mobile, and non-tender. It emerged from the pelvis because its lower border was inaccessible. Per-speculum vaginal examination revealed a healthy cervix and vagina. There was a mass that corresponded to 28 weeks of the pregnant uterus. It was not separate from the uterus. Bilateral adnexa was clear. She had been a known case of hypertension for three years and was on medications. Investigations were carried out as per Table 1.

**Table 1:** Investigations Hb: Hemoglobin; RBC: Red blood cell; WBC: White blood cell; DLC: Differential leukocyte count; PT: Prothrombin time; INR: International normalized ratio; ALT: Alanine aminotransferase (also known as glutamate pyruvate transaminase (GPT)); AST: Aspartate aminotransferase (also known as glutamic oxaloacetic transaminase (GOT)); LFT: Liver function test; FBS: Fasting blood sugar; PMBS: Postprandial blood sugar; M: Male; F: Female; Ped: Pediatric

Investigation	Values	Reference values
Hb%	12.3 gm%	M: 13-15.5 gm%; F: 12-14.5 gm%
Total RBC count	6.08 million/cu.mm	M: 4.5-6 million/cu.mm; F: 4.5-6 million/cu.mm
Total WBC count	9200/cu.mm	M and F: 4000-11000/cu.mm
DLC - polymorphs	62%	M: 40-80%; F: 40-80%; Ped: 15-80%
DLC - lymphocytes	30%	M: 10-20%; F: 10-20%; Ped: 15-20%
PT (INR and index)	15.3 sec	Control: 13.0 sec
ALT (GPT)	12.9 IU/L	M (adult): <50 IU/L; F (adult): <35 IU/L
AST (GOT)	15.4 IU/L	M (adult): <50 IU/L; F (adult): <35 IU/L
Alkaline phosphatase	56.8 IU/L	30-120 IU/L
Protein - serum	7.17 g/dL	6.6-8.3 g/dL
Bilirubin - total	0.43 mg/dL	0.3-1.2 mg/dL
Creatinine - serum	0.9 mg/dL	M: 0.72-1.18 mg/dL; F: 0.55-1.02 mg/dL
Potassium (K+) - serum	3.2 mEq/L	3.5-5.1 mEq/L
Sodium (Na+) - serum	137 mEq/L	136-146 mEq/L
Urea - serum	37.73 mg/dL	17-43 mg/dL
Pus cell	Occasional	NA
FBS	88 mg/dL	70-100 mg/dL
PMBS	138 mg/dL	<140 mg/dL

**Figure 1:**
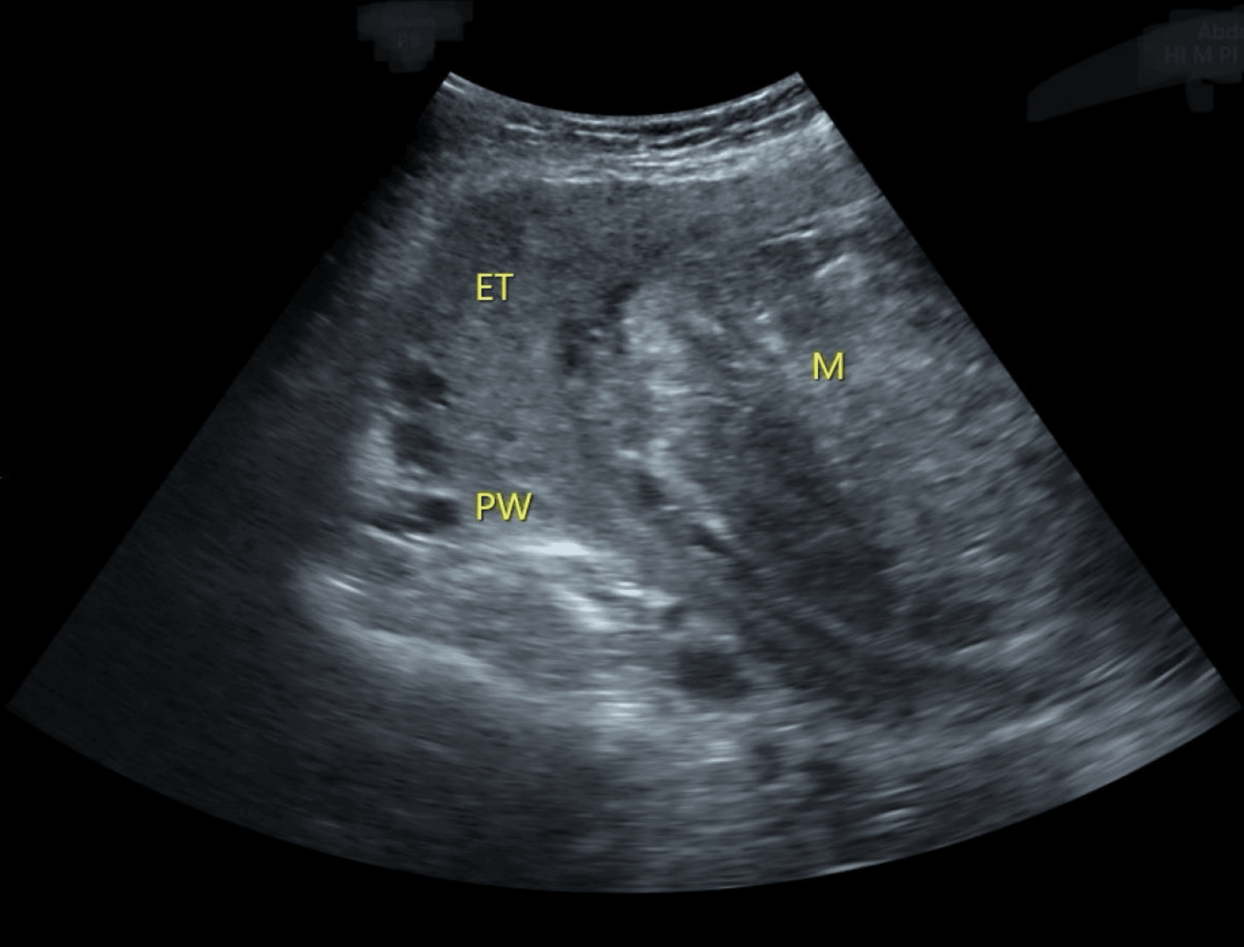
Ultrasound image showing huge fundal fibroid arising from the anterior wall of the uterus M: Mass; PW: Posterior wall of the uterus; ET: Endometrial thickness

Ultrasonography showed a large fibroid 10.2 × 11.7 × 2.8 cm. The uterus was bulky 17 × 10.9 × 12 cm, and the ovaries were normal, as shown in Figure 1.

**Figure 2:**
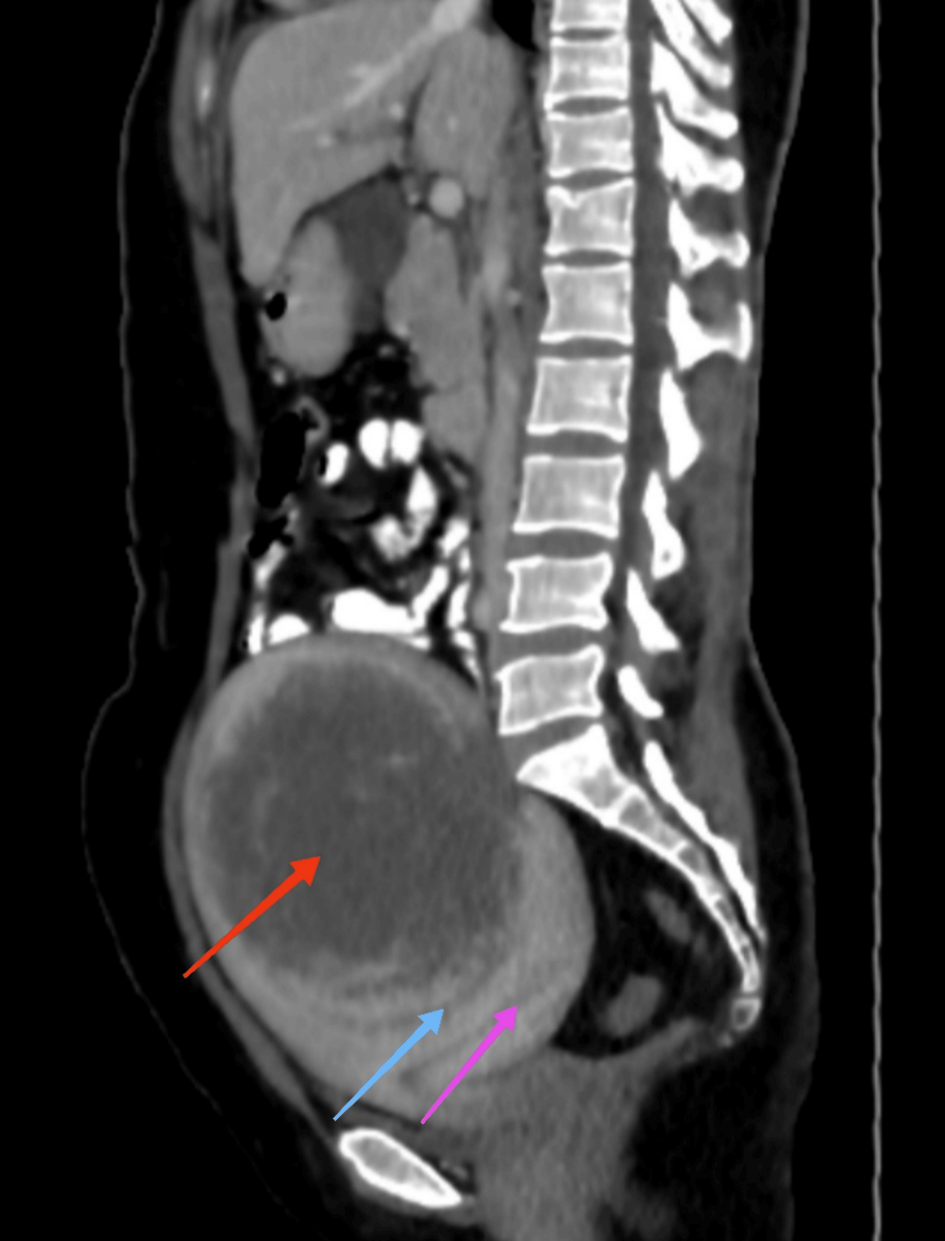
Computed tomography scan in sagittal section showing huge fundal fibroid arising from the anterior wall of the uterus The red arrow shows the mass, the blue arrow shows the capsule of the fibroid, and the pink arrow shows the endometrial cavity

A CT scan showed a large uterus, with a single big mass suggestive of leiomyoma, as shown in Figure 2.

**Figure 3:**
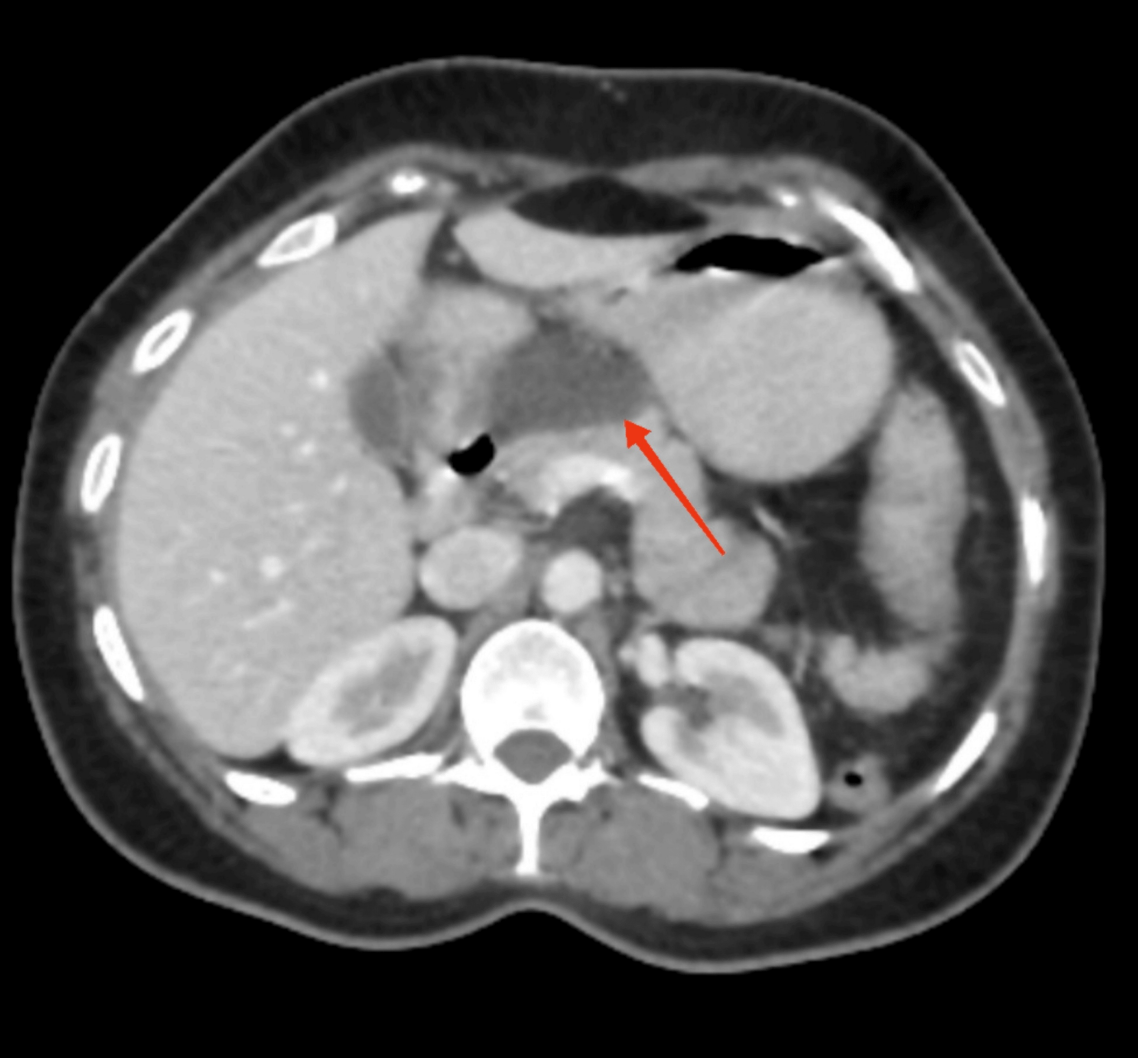
Computed tomography scan in coronal view showing the peripancreatic cyst The red arrow shows the peripancreatic cyst

There was a hypodense collection of 3.8 × 1.4 cm in the lesser sac, which was a peri-pancreatic benign cyst suggestive of lymphangioma, as depicted in Figure 3.

**Figure 4:**
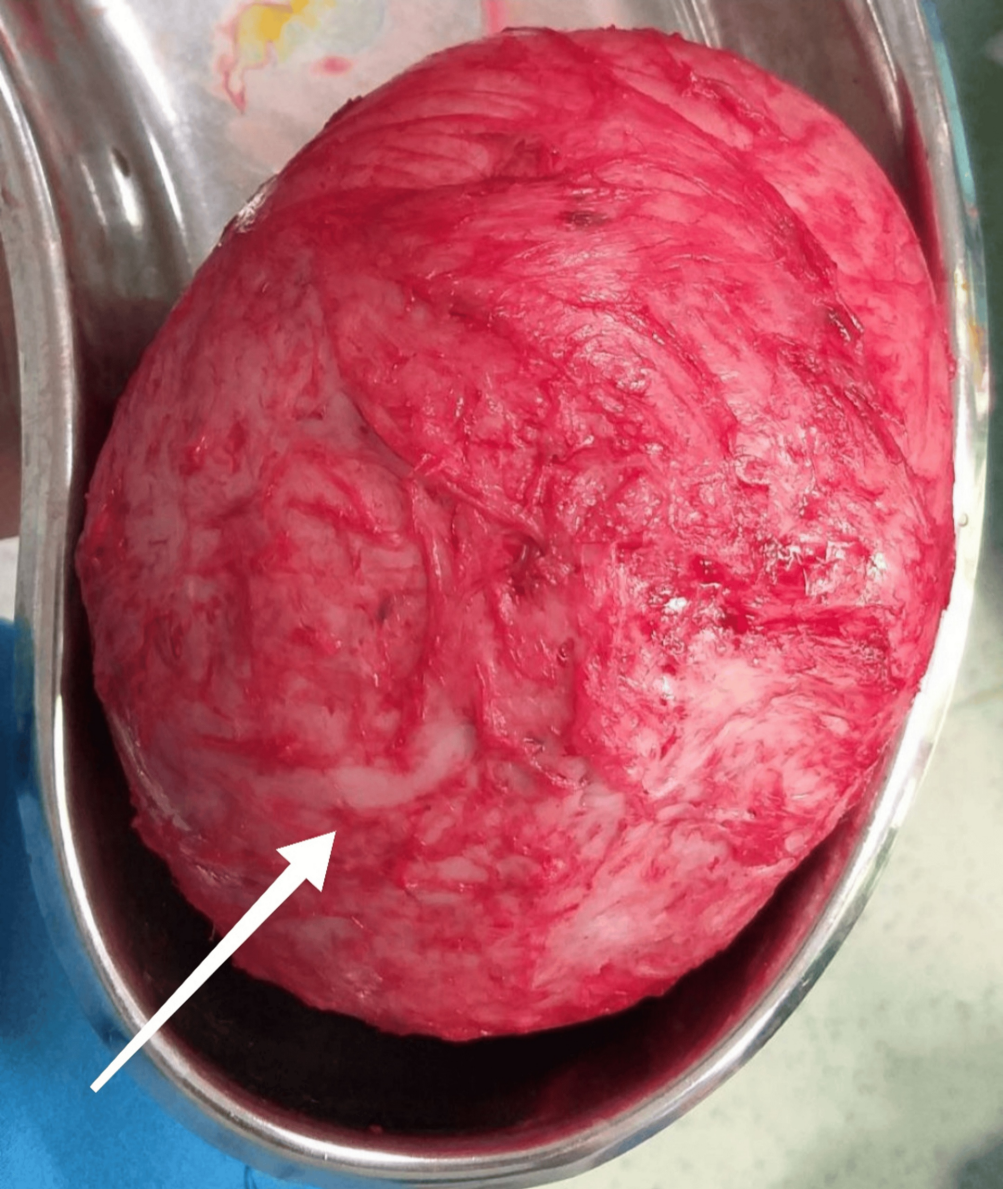
Encapsulated myomectomy specimen weighing 2.5 kg and measuring 12 × 10 × 8 cm The white arrow shows the myoma

The CA-125 was normal. The patient underwent evaluation under anesthesia (EUA). The findings confirmed a uterocervical length (UCL) of 3.5 cm. Endometrial curettage was performed. Histopathology report (HPE) showed endometrium in the late secretory phase. She underwent myomectomy. Intraoperatively, a single huge fibroid corresponding to 28 weeks was seen arising from the fundus. No breach of the capsule was found, and the vascularity was not increased. Both ovaries and tubes were normal. The cavity of the uterus was not opened. She received two units of blood transfusion. Myomectomy was uneventful, and postoperative recovery was good. The myoma removed was 2.5 kg and measured 12 × 10 × 8 cm, as shown in Figure 4.

**Figure 5:**
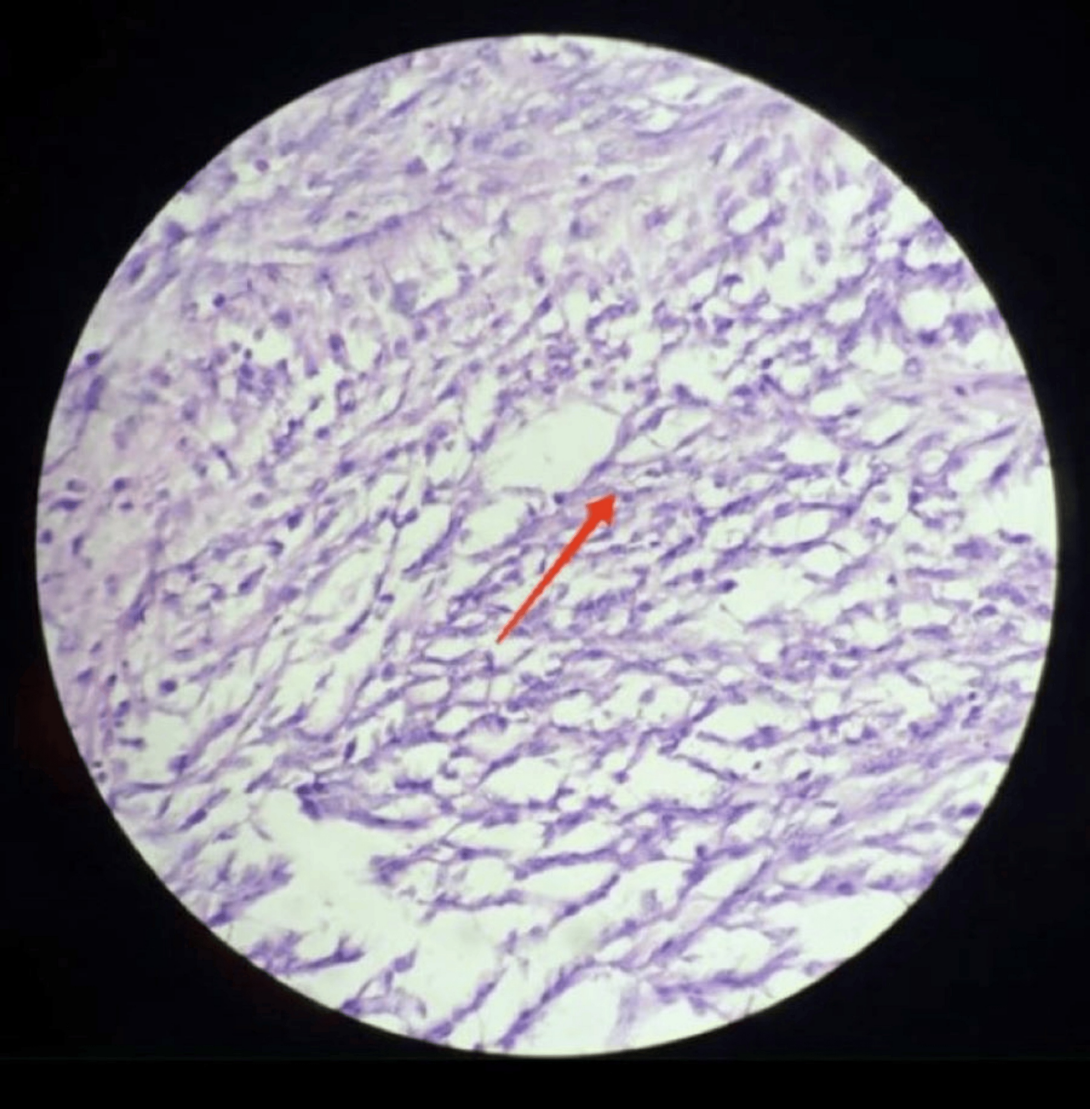
Individual spindle-shaped tumor cells having pleomorphic vesicular hyperchromic nuclei The red arrow shows spindle-shaped tumor cells

HPE reported it as LMS, with five to six mitotic figures per high-power field and proliferative blood vessels. Tumor cells were arranged in interlacing fascicles under a high-power field. Individual cells are spindle-shaped, having pleomorphic vesicular hyperchromic nuclei under a 40-high-power field, as shown in Figure 5.

After the oncologist's opinion, total abdominal hysterectomy (TAH) and bilateral salpingo-oophorectomy (BSO) with omentectomy were performed. Intraoperatively, there were adhesions between the fundus and the small bowel. Intraoperative and postoperative periods were normal. The HPE was normal, and the myometrium showed a foreign body giant cell reaction. She was discharged on the 11th postoperative day after stitch removal. She was advised of chemotherapy. The patient remained disease-free for seven months, and she was started on palliative chemotherapy.

## Discussion

Uterine sarcoma is a highly malignant and extremely aggressive tumor that develops from the smooth muscles of the uterus. The aggregate risk of LMS is 1.78 per 1,000 or 1 in 562. The aggregate risk of other uterine sarcomas is 1.16 per 1,000 or 1 in 861. The overall risk of uterine sarcoma is 2.94 per 1,000 or 1 in 340. The prediction of the risk of uterine sarcoma after age stratification ranges from a peak of 10.1 cases per 1,000, or 1 in 98, for patients aged 75-79 years to <1 case per 500 for patients aged <30 years [10].

Tumors are most often diagnosed in the age group of 35-75 years, with a spike in incidence during the perimenopausal years [4]. The age-specific incidence of LMS shows a peak at menopause [11]. Our patient was only 35 years old. As the doubling time of this tumor is four weeks, it is rapidly growing and aggressive [8]. Uterine sarcomas are usually diagnosed in post-menopausal women between the ages of 50 and 70 years [5]. It is an extremely aggressive malignancy associated with a poor overall prognosis. LMS has no specific symptoms, so diagnosing it before operation is challenging. The appearance of LMS and fibroids mimic each other [12]. Sixty percent of patients are diagnosed at an early stage. Nevertheless, the prognosis for LMS is dismal regardless of the stage [13].

It is still challenging to diagnose uterine sarcoma before surgery. To date, benign myomas and malignant sarcomas cannot be distinguished by imaging modalities reliably. While not accurate, MRI might offer some information. Uterine LMS are extremely aggressive and have a high rate of recurrence. In our case, histological examination was used to diagnose uterine sarcoma. TAH and BSO are the usual surgical procedures for treating uterine sarcoma. Since less than 3% of cases involve lymph nodes, pelvic and para-aortal lymph node dissection is not advisable. Prognostic indicators are tumors larger than 5 cm and a high mitotic index. A tumor with a mitotic count of less than 2 per square millimeter is also considered aggressive. The commonest mode of spread is hematogenous, while lymphatic spread is uncommon. After surgery, the patient requires multidisciplinary care involving a gynecologist, an oncologist, an oncologist chemotherapist, and a radiologist. The rate of recurrence in stages I and II is around 70%. The lungs and upper abdomen are the most common sites of recurrence [6-8]. The stage of disease at diagnosis is the most important factor for survival. The five-year survival rate is 50-55% for stage I and 8-12% for stages II-IV [7]. On average, the five-year survival rate for all stages is between 30 and 50%. Surgery can be done for local recurrences. Resection can be done for isolated pulmonary metastasis. The overall 5-year and 10-year survival rates for local recurrence are 45 and 35%, respectively, in cases of pulmonary metastasis.

## Conclusions

LMS is a rare but extremely aggressive malignancy of perimenopausal females that has a very poor prognosis and a high mortality rate. It is difficult and challenging to detect it preoperatively as they have no specific signs and symptoms. CT scans cannot differentiate benign and malignant tumors. As the screening of LMS is not possible due to its rarity, early diagnosis and management are of utmost importance. It should be immediately treated by TAH and BSO, the only surgical treatment, as its doubling time is about four weeks. Future studies on preoperative diagnostic tests and variables affecting prognosis should be undertaken to improve the overall survival outcome.
